# Comparison of Metabolic Syndrome, Autoimmune and Viral Distinctive Inflammatory Related Conditions as Affected by Body Mass Index

**DOI:** 10.3390/jcm13216298

**Published:** 2024-10-22

**Authors:** Lourdes Chero-Sandoval, María Martínez-Urbistondo, Amanda Cuevas-Sierra, Andrea Higuera-Gómez, Eva Martin-Domenech, Raquel Castejón, Susana Mellor-Pita, Víctor Moreno-Torres, Omar Ramos-Lopez, Daniel de Luis, Juan Antonio Vargas, J. Alfredo Martínez

**Affiliations:** 1 Precision Nutrition and Cardiometabolic Health, IMDEA-Food Institute (Madrid Institute for Advanced Studies), Campus of International Excellence (CEI) UAM+CSIC, 28049 Madrid, Spain; lourdeschero88@gmail.com (L.C.-S.); andrea.higuera@alimentacion.imdea.org (A.H.-G.); 22289913@live.uem.es (E.M.-D.); jalfredo.martinez@imdea.org (J.A.M.); 2 Endocrinology and Nutrition Department, Clinical University Hospital of Valladolid, 47003 Valladolid, Spain; danielantonio.luis@uva.es; 3 Internal Medicine Service of the Puerta de Hierro Majadahonda University Hospital, 28222 Madrid, Spain; mmurbistondo@gmail.com (M.M.-U.); susanateresa.mellor@uam.es (S.M.-P.); victor.moreno.torres.1988@gmail.com (V.M.-T.); juanantonio.vargas@salud.madrid.org (J.A.V.); 4 UNIR Health Sciences School and Medical Center, Universidad Internacional de la Rioja, 26004 Madrid, Spain; 5 Medicine and Psychology School, Autonomous University of Baja California, Tijuana 22390, Mexico; oscar.omar.ramos.lopez@uabc.edu.mx

**Keywords:** health markers, inflammation, long COVID, obesity, systemic lupus erythematosus

## Abstract

**Background**: Metabolic inflammation (MI), long COVID (LC) and systemic lupus erythematosus (SLE) share some metabolic common manifestations and inflammatory pathophysiological similarities. Health-related quality of life (HRQoL) and metabolic age are indicators of health status. The “METAINFLAMMATION-CM Y2020/BIO-6600” project, a prospective controlled study, aimed to identify differential diagnostic tools and clinical features among three inflammatory conditions by comparing obesity status (low BMI vs. high BMI). **Methods**: A total of 272 adults of both Caucasian and Hispanic descent, diagnosed with MI, LC or SLE, and a range of BMI, were recruited. Clinical and phenotypic traits were measured to analyze body composition, metabolic and inflammatory markers, HRQoL data, metabolic age and lifestyle habits using a 3 × 2 (disease × BMI) factorial design. **Results**: Some inflammatory related variables, such as fibrinogen, RDW (red cell blood distribution width), ESR (erythrocyte sedimentation rate) and NLR (neutrophil/lymphocyte ratio), showed effect modifications depending on the BMI and disease type. In relation to HRQoL, the Physical Component Summary (PCS12) showed no relevant changes, while the Mental Component Summary (MCS12) showed a significant effect modification according to the disease type and BMI (*p* < 0.05). Furthermore, a significant interaction was identified between the disease type and BMI in relation to metabolic age (*p* = 0.02). **Conclusions**: Assessing the impact of BMI on these three inflammatory diseases may help to prevent clinical complications and to design personalized treatments, especially for patients with SLE, who have a worse prognosis with an increased BMI compared to the other two inflammatory diseases.

## 1. Introduction

Inflammation is an adaptive pathophysiological component of the immune system to fight against adverse agents such as infection or injury [1]. Symptoms including redness, swelling, heat, pain, and loss of function are induced by complex biological processes, which may differ among inflammatory related diseases [2]. Furthermore, inflammation can manifest acutely or chronically depending on endogenous and exogenous factors and mediators [3]. In acute inflammation, there is an immediate response to recruit immune cells and combat the threat [1,4]. When the underlying cause is removed, the inflammation ceases, and the integrity of the tissue is usually recovered [4]. However, in chronic inflammation, outcomes become persistent and can cause irreversible tissue damage [5]. Chronic inflammation is related to several prevalent diseases, such as obesity, cardiovascular events, viral infections, autoimmune diseases and cancer [2,6]. In this context, metabolic inflammation (MI), long COVID (LC) and systemic lupus erythematosus (SLE) are different proinflammatory pathological conditions that share common metabolic manifestations with pathophysiological and clinical similarities, such as chronic inflammation, immune dysfunction and risk of parallel complications, but remain different in etiological origin, severity and duration and management possibilities [7,8,9,10,11,12].

Inflammatory status has been assessed through the measurement and analysis of different mediators and proxies [13]. Thus, diverse inflammatory biomarkers elicit different responses depending on the inflammatory type, such as interleukin-6 (IL-6) or tumor necrosis factor-alpha (TNF-α), have been associated with the inflammation process, but are not routinely assessed [13,14]. On the other hand, the concentrations of C-reactive protein (CRP) can vary under diverse insults, such as bacterial infections, tissue damage, cardiovascular alterations and autoimmune diseases, and can also be a prognostic factor [15]. The neutrophil/lymphocyte ratio (NLR) is a practical tool obtained from peripheral blood analysis able to monitor inflammatory outcomes [16]. In addition, the red cell distribution width (RDW) calculated on a complete blood count provides information on the variability of circulating red cell size, which is related to inflammation [17]. Several trials have demonstrated the preeminent role of fibrinogen as a marker of inflammatory disease as exerting pleiotropic effects through multiple targets, substrates and immune mechanisms [18]. Finally, erythrocyte sedimentation rate (ESR) is a laboratory test used to confirm morbid conditions, accompanying inflammatory status [19]. Indeed, these determinants contribute to feature inflammation but all of them are needed to develop an integrated inflammation vision.

In this context, health, defined as physical, mental, and social well-being, is complemented by the subjective perception of one’s quality of life, considering personal goals and concerns [20]. Health-related quality of life (HRQoL) functions as a health indicator for evaluating the consequences of illness and therapy. This evaluation helps assess healthcare interventions and risks associated with chronic inflammation. Thus, the validated Medical Outcomes Study Short-Form-12 (SF-12) questionnaire evaluates quality of life by measuring the Physical Component Summary (PCS12) and Mental Component Summary (MCS12), with the values to indicate the health status estimation [21,22]. Also, metabolic age is a surrogate including health status [23]. Each of the three diseases (MI, LC and SLE) presents a high degree of heterogeneity in terms of pathophysiology and clinical presentation. Comparing these three medical conditions is critical, although chronic inflammation is a common feature of many diseases, the severity of symptoms and clinical manifestations vary considerably, ranging from mild metabolic inflammation to severe SLE. Therefore, there is a need for deep phenotyping and to develop personalized tools that contribute to understanding interindividual clinical differences among inflammatory conditions for early diagnosis, for prevention of complications and for precision management [24,25].

This research aims to identify the body mass index (BMI) influence on inflammatory outcomes and clinical status in patients with MI, LC and SLE by analyzing the relationship between BMI and clinical and metabolic markers in these three chronic inflammatory conditions. Indeed, it was focused on understanding the differential impact of excess body weight on inflammation and the differentiated and specific risks associated with each disease.

The choice of these three conditions is justified by their similarities and differences in inflammatory and immunological mechanisms. Obesity, recognized as a cardiovascular risk factor, not only affects patient prognosis, but also generates diverse clinical outcomes and alters response to treatment as a low-grade inflammatory state [26]. Moreover, BMI is a significant risk factor in the progression of metabolic syndrome, lupus and COVID-19 manifestations [26,27,28,29]. Additionally, associated comorbidities such as hypertension and dyslipidemia can further complicate the management of these conditions [30]. From a translational approach, the study aims to improve diagnostic accuracy and therapeutic management by using routine inflammatory markers, which will allow the personalization of treatments and reduce overweight-related complications commonly interacted in patients suffering an inflammatory phenomenon [31,32]. Taken together, these findings could optimize clinical strategies for better management of chronic inflammation in patients with MI, LC and SLE, where immunocompetence may be compromised in all three conditions [24,26,33,34].

## 2. Materials and Methods

### 2.1. Study Design

This research belongs to the “METAINFLAMMATION-CM Y2020/BIO-6600” project, which is a prospective and controlled study. Participants were recruited between January 2022 and June 2023, at the Internal Medicine Service of the Puerta de Hierro Majadahonda University Hospital (Madrid, Spain). This study was carried out in accordance with the Declaration of Helsinki and was approved by the Research Ethics Committee of the Puerta de Hierro Majadahonda University Hospital with reference (PI 164-21). The volunteers signed a written informed consent form before their inclusion in the study. Anthropometric measurements and body composition, validated lifestyle questionnaires and biochemical and HRQoL variables were analyzed according to the approved ethical criteria and protocols of the hospital.

### 2.2. Study Participants: Inclusion and Exclusion Criteria

The participants were categorized into three groups for analysis: MI, LC and SLE. The MI group included patients presenting with a combination of obesity and metabolic syndrome (MetS), considered to be manifestations of grade inflammation [35]. Obesity is described as an excessive accumulation of body fat, usually caused by a prolonged positive energy balance and a sedentary lifestyle [35,36], while MetS, according to the World Health Organization (WHO), is defined by the presence of abdominal obesity, insulin resistance, hypertension and hyperlipidemia [37], and is associated with an increased risk of developing type 2 diabetes, obesity and atherosclerosis [38,39]. The criteria established by the WHO and National Cholesterol Education Program (NECP), Cholesterol Treatment Panel. Adults III (ATP III) [40] for obesity and metabolic syndrome, were used to identify these patients [32]. The LC group was established with patients who had pathophysiological inflammation underlying a viral infection [41] and followed the guidelines of the National Institute for Health and Care Excellence (NICE) and National Institute for Health and Care Research (NIHR) [42,43]. Finally, the SLE group consisted of patients with a disease characterized by chronic activation and inflammation of the immune system, who met the classification criteria established by the European League Against Rheumatism/American College of Rheumatology for SLE [44].

#### 2.2.1. Inclusion Criteria

Study participants met the following specific inclusion criteria: age > 18 years old, a BMI > 17.01 kg/m^2^ and <51.35 kg/m^2^, and diagnosis of MI, LC and SLE according to previous description and confirmed by the medical staff of the Internal Medicine service at the Puerta de Hierro Majadahonda University Hospital (Madrid, Spain).

First, patients with obesity and metabolic syndrome and various clinical alterations were observed, such as excessive adiposity, glucose intolerance, central obesity, dyslipidemia, and hypertension [40]. On the other hand, LC patients were those who presented persistent symptoms related to COVID-19 infection beyond 4 weeks after the onset of initial acute symptoms. These symptoms included constant fatigue, shortness of breath, muscle or joint pain, among others, with a significant impact on their quality of life [41]. To avoid bias in the evaluation, patients with SLE in a stable state, with controlled symptoms and under adequate medical treatment, were selected. In these patients, clinical parameters such as serological activity (SA), the presence of active disease (AD), the achievement of complete remission (CR) and the maintenance of a low disease activity state (LDAS) were evaluated. Anti-dsDNA antibody levels treatments received, including steroids alone, steroids combined with other immunosuppressants or nonsteroidal immunosuppressive drugs, were also considered [45,46,47]. SLE disease activity was measured using the SLE Disease Activity Index (SLEDAI-2k), while organ damage was assessed using the SLICC/American College of Rheumatology (ACR) tool [46,47].

#### 2.2.2. Exclusion Criteria

Exclusion criteria included the presence of severe psychiatric disorders, the current use of body weight-modifying agents, lack of independence, inability to diet, difficulty in scheduling appointments and pregnancy or lactation.

### 2.3. Variables Analyzed

#### 2.3.1. Anthropometric and Body Composition Measurements

The anthropometric and body composition measurements were collected at baseline in the Internal Medicine Department of the Puerta de Hierro Majadahonda University Hospital by a trained dietitian using validated methods [48]. Body weight including total muscle mass, total fat mass, visceral fat and metabolic age were assessed using the bioimpedance equipment scale (TANITA SC-330; Tanita Corporation, Tokyo, Japan), which also estimated body composition. Waist was measured using a commercial tape measure following validated protocols [49]. BMI was calculated as body weight divided by squared height (kg/m^2^) [48] and using the international WHO criteria (BMI at normal weight <24.9 kg/m^2^; BMI at overweight <29.9 kg/m^2^; BMI in obesity ≥30 kg/m^2^) [32].

#### 2.3.2. Hematological and Biochemical Measurements

Blood samples were collected in fasting conditions by venipuncture following validated hospital protocols. These sanguineous samples were analyzed for leukocytes, lymphocytes, neutrophils, platelets, ESR and RDW determinations in the hematology laboratory of the Puerta de Hierro Majadahonda University Hospital, using a SYSMEX XN-20 automated hematology analyzer (Roche, Basel, Switzerland). The NLR was calculated directly from the measured values [48]. The reference range for RDW in the hematology laboratory of the hospital is 8–14.8%, with values above 14.8% being considered pathological [50].

The routine biochemical markers such as glucose, total cholesterol, ferritin, triglycerides, uric acid, alanine aminotransferase (ALT), aspartate aminotransferase (AST) and transferrin were performed following standardized hospital protocols with equipment meeting accredited criteria in a quality controlled autoanalyzer (Atellica™ Solution Pais) as described elsewhere [51]. Variables related to prognosis, proinflammatory factors and markers such as CRP, fibrinogen, insulin, lactate dehydrogenase (LDH), D-dimer, N-terminal pro-B-type natriuretic peptide (NT ProBNP), IL-6 and prothrombin activity also followed standardized procedures mainly with ELISA kits (Sigma-Aldrich ELISA Kit, St. Louis, MO, USA) as described by the suppliers.

#### 2.3.3. Clinical Metabolic Measurements and Quality of Life Data

Systolic and diastolic blood pressures were measured with a sphygmomanometer, following standardized criteria based on international guidelines [31]. Patients completed questionnaires related to sociodemographic data, metabolic history, lifestyle (physical activity, sleep habits and nutrition) and HRQoL guided by a trained dietitian. Metabolic age was assessed based on sex, body composition and metabolic rate data (TANITA SC-330; Tanita Corporation, Tokyo, Japan) [52], while the HRQoL was accounted through a validated instrument: the SF-12 questionnaire, which assesses both the PCS12 and MCS12 health, uses a rating scale from 0 to 100 [53] and is applicable in patients with chronic inflammatory characteristics such as obesity, MetS, LC and SLE [54,55,56,57,58].

### 2.4. Statistical Analyses

Variables were expressed as means ($\bar{x}$) and standard deviations (SDs) for quantitative variables and number of cases (n) and percentage (%) for qualitative variables. Normality of the data was assessed by Shapiro–Wilk test. Student’s t tests were mostly implemented to compare the means of the continuous variables at the beginning of the study and the categorical variables were statistically analyzed using the Chi-square (χ^2^) test. The differences and interactions between the three types of diseases and the BMI stratified by p50 were studied with a 3 × 2 factorial analysis of variance (ANOVA) design (3 diseases × 2 levels of BMI) concerning the anthropometric variables, body composition, biochemical, HRQoL and inflammatory status features involving METAINFLAMMATION study participants.

To illustrate the findings, two types of graphs were created. The first was a bar graph representing the mean of inflammatory variables, such as fibrinogen, RDW, ESR and NLR as a function of a dichotomised BMI for each disease. The bar graph of the means followed the “per protocol” (PP) approach, while the simple linear regression illustration followed the “intention to treat” (ITT) approach. The second graph, a simple linear regression model, showed the interaction between the three groups of chronic inflammatory state and BMI (lower or higher) in relation to PCS12, MCS12 and metabolic age was evaluated.

Furthermore, we assessed the three inflammatory disease groups, considering additional variables such as quality of life and metabolic age. Finally, the regression model was constructed using lymphocytes, NLR, RDW, ESR, fibrinogen, PCS12 and MCS12, as dependent variables and the age, sex, BMI, an interaction between disease and BMI at levels (lower and higher) as independent variables.Results were presented as regression coefficients (β), 95% confidence intervals and *p*-values, with a significance level of *p* < 0.05. To carry out all these analyses, R Studio software (version 4.2.2) was used, which facilitated the handling of the data and the execution of the necessary statistical tests. Overall, this rigorous methodological approach allowed researchers to gain a clearer understanding of the complex interactions between BMI and inflammatory diseases, thus contributing to suitable medical translation.

## 3. Results

### 3.1. Assessment of Anthropometric Measurements, Biochemical Data and Quality of Life Questionnaires at Baseline

The total population (n = 272) of adults of both Caucasian and Hispanic descent, with a gender distribution of 22% men and 78% women, was first categorized by the physicians into three groups of disease (MI, LC and SLE) according to the medical diagnosis. Subjects with obesity and metabolic syndrome were grouped under the term metabolic inflammation. Individuals were further sub-classified according to the median BMI, with “lower BMI” (for those with values below 28.7 kg/m^2^) or “higher BMI” (for those with values above 28.7 kg/m^2^ up to 51.3 kg/m^2^).

The comparisons of anthropometric, body composition, biochemical and quality of life data depending on each disease and the BMI status at baseline were reported (Table 1). As expected, all groups demonstrated significant differences in such variables when categorized by BMI values (*p* < 0.001). In every inflammatory diseased group, waist circumference (*p* < 0.001), fat mass (*p* < 0.001), visceral fat (*p* < 0.001) and metabolic age (*p* < 0.001) evidenced remarkably higher values in patients with a higher BMI. In addition, both components of HRQoL (PCS12 and MCS12, *p* < 0.001) were significantly lower in patients with a higher BMI, except in the SLE group, where patients with a higher BMI presented slightly higher values in MCS12. Also, in the MI group, patients with an increased BMI presented elevated values of insulin (*p* = 0.004).

In the LC group, patients with higher a BMI showed higher values of SBP (*p* < 0.001), glucose levels (*p* = 0.001), insulin (*p* < 0.001), triglycerides (*p* < 0.001) and the AST/ALT ratio (*p* < 0.001). In addition, the SLE group with a high BMI presented statistical differences in blood pressure (SBP and DBP, *p* = 0.003 and *p* = 0.04 respectively) (Table 1).

Also, the ANOVA analysis (Group column, Table 1) revealed that all variables were significantly different between all groups of diseases, except transferrin and insulin (without considering BMI status). These outcomes highlight that the MI group presented higher values for these anthropometric and biochemical measurements. Interestingly, the LC group presented the highest values of sadness (Table 1). When the variables were compared by BMI status (without considering type of disease), all variables were significantly different, excepting age, MCS12, prothrombin activity and sadness level (Table 1).

The interactions between the type of disease and the BMI (Interaction column, Table 1) were statistically significant in age (*p* = 0.01), waist circumference (*p* = 0.01), total fat mass (*p* = 0.01), metabolic age (*p* = 0.02), SBP (*p* = 0.01) and MCS12 (*p* < 0.05), indicating relevant effect modification concerning disease type and as a function of the BMI.

### 3.2. Assessment of Hematological and Inflammatory Variables

Variables related to inflammatory status were reported, which were displayed according to group of disease and stratified by BMI status (Table 2). In the MI group, patients with a higher BMI showed increased values of uric acid levels (*p* < 0.001). Also, in the LC group, patients showed significant differences in uric acid levels compared by BMI status. Interestingly, subjects with LC and a higher BMI presented significantly higher values of LDH (*p* = 0.01), CRP (*p* = 0.003), blood leukocytes (*p* = 0.02), platelets (*p* = 0.02), ESR (*p* = 0.03) and fibrinogen (*p* = 0.01). In the SLE group, patients with a higher BMI presented significantly higher values of uric acid (*p* = 0.04), LDH (*p* = 0.01), ESR (*p* = 0.004) and fibrinogen (*p* = 0.001). Also, patients with SLE presented lower levels of lymphocytes (*p* = 0.04).

When the values were compared by the type of inflammatory disease (Group column, Table 2), significant differences (*p* < 0.05) were found in uric acid, CRP, ferritin, lymphocytes, NLR, RDW, ESR and fibrinogen. Higher values for these variables were observed in the SLE group, especially for NT proBNP, CRP, NLR, RDW, ESR and fibrinogen, suggesting high status of inflammation in this disease. While values were compared by BMI status (without considering type of disease), differences (*p* < 0.05) were found in uric acid, LDH, CRP, leukocytes, NLR, RDW, ESR and fibrinogen, showing higher values for these variables in subjects with a higher BMI.

The interaction analysis showed significant effect modification (3 diseases × 2 levels of BMI) differences in uric acid levels, lymphocytes, NLR, ESR and fibrinogen, indicating that these variables presented differences not only depending on the type of disease, but are also differently affected depending on the BMI.

### 3.3. Bar Graphs Representation and Simple Linear Models Concerning Inflammatory Variables Depending on Disease and BMI

**Figure 1:**
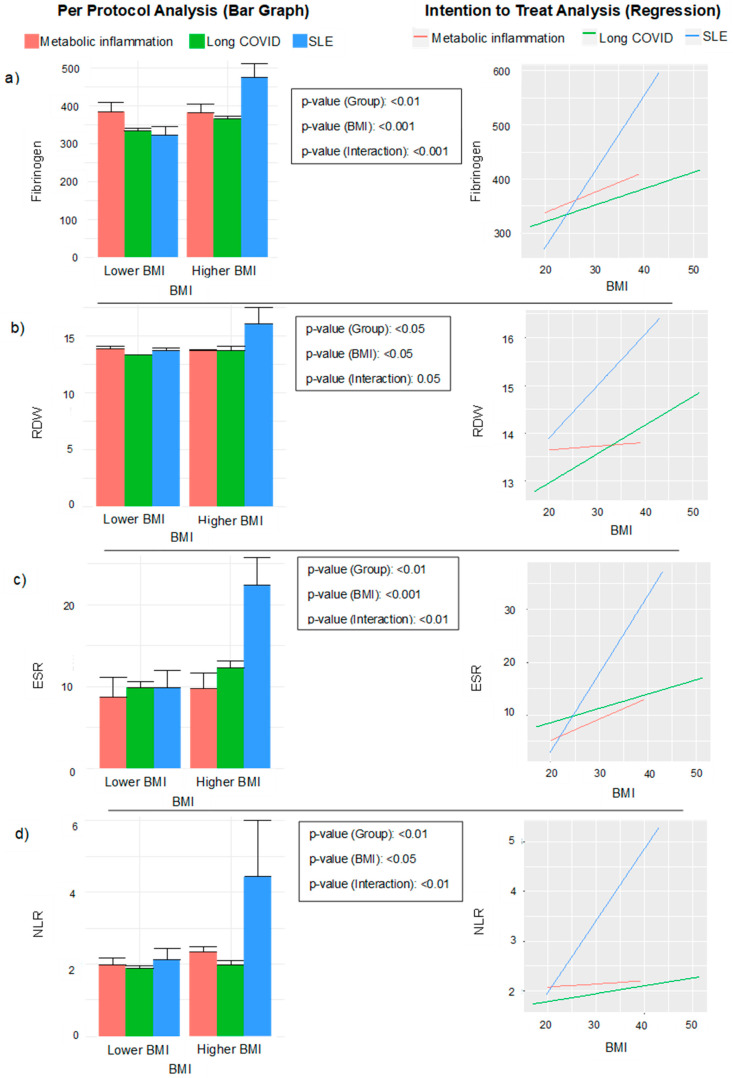
Distribution of disease groups based on body mass index (kg/m^2^) in relation to (**a**) fibrinogen, (**b**) red cell distribution width (RDW), (**c**) erythrocyte sedimentation rate (ESR) and (**d**) neutrophil-lymphocyte ratio (NLR).

In Figure 1a, the left bar graph concerning the BMI and the disease groups illustrating fibrinogen data shows that there were significant differences between the groups (*p* < 0.01), among the BMI categories (*p* < 0.001) and an interaction between the inflammatory state and dichotomized BMI (*p* < 0.001) when using the per protocol (PP) approach. On the other hand, after adjusting the right linear model (Figure 1a) using the intention to treat (ITT) model, it was found that the lower the BMI, the lower the amount of fibrinogen for SLE, slightly higher levels observed for LC and metabolic inflammation. Indeed, when the BMI increased, fibrinogen levels augmented considerably for SLE, but less for the other conditions.

The second left graph concerns the RDW data (Figure 1b) following PP criteria, which shows that there were relevant differences between the groups of disease (*p* < 0.05) and among the levels of the BMI (*p* < 0.05) and the interaction between the type of disease and the BMI (*p* < 0.01). The right linear model (Figure 1b) based on ITT analysis revealed that the higher the BMI, the higher the RDW level for LC, with slightly higher levels observed for metabolic inflammation and SLE. Interestingly, when the BMI increased, the amount of RDW increased at a greater extent for SLE again.

Considering the ESR, the left bar graph (Figure 1c) with PP analysis shows that there were significant differences between the groups of disease (*p* < 0.01), depending on the BMI status (*p* < 0.001) and the interaction between the type of disease and the BMI (*p* = 0.05). Likewise, the right linear model under the ITT approach (Figure 1c) shows that the lower the BMI, the lower the ESR for SLE followed by MI and LC, while, when the BMI increased, the ESR for SLE also increased.

Finally, the left bar graph for the NLR (Figure 1d) following PP criteria demonstrates significant differences between the groups of disease (*p* < 0.01), among the BMI subgroups (*p* < 0.05) and between the interaction with the type of disease and the BMI status (*p* < 0.01). Furthermore, the right linear model based on ITT analysis (Figure 1d) evidenced that the lower the BMI, the lower the NLR for LC followed by SLE, whereas, when the BMI increased, the NLR was considerably elevated for SLE.

The bar graphs and graphic visualization of the linear models of (Figure 1a) fibrinogen, (Figure 1b) RDW, (Figure 1c) ESR and (Figure 1d) and NLR were compared by the type of disease and by BMI status (lower and higher) for each disease group (MI, LC and SLE), using per protocol and intentions to inform treatment approaches. BMI, Body Mass Index; ESR, erythrocyte sedimentation rate; LC, long COVID; MI, metabolic inflammation; NLR, neutrophil/lymphocyte ratio; RDW, red cell blood distribution width; SLE, systemic lupus erythematosus.

### 3.4. HRQoL and Metabolic Age Analysis by Dichotomized BMI

**Figure 2:**
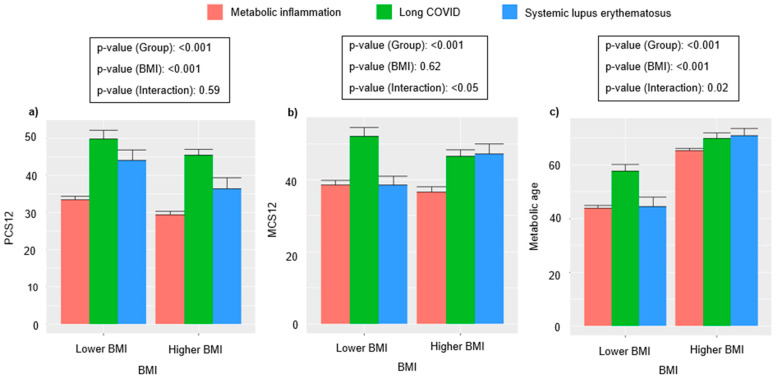
Impact of dichotomized BMI on HRQoL and metabolic age. (**a**) on physical component from HRQoL (PCS12), (**b**) on mental component from HRQoL and (**c**) on metabolic age.

The dichotomized BMI distribution and the three inflammatory groups were analyzed in relation to PCS12 (Figure 2a). This analysis evidenced that there was an inverse relationship between BMI and PCS12 values for MI, LC and SLE. Thus, when the BMI increased, the PCS12 values decreased in this population. When the relationship between BMI and MCS12 was analyzed, an interaction between BMI and disease group was found (Figure 2b), showing that MCS12 was higher when the BMI was lower, while when the BMI increased; the mean value of MCS12 decreased for LC and MI patients. Interestingly, the values of MCS12 were higher for patients with SLE and a higher BMI. The third graph in relation to metabolic age (Figure 2c) illustrates that when the BMI is lower, the mean value of the metabolic age is reduced for every group of inflammatory disease, showing a direct relationship.

The bar graphs show the interactions between the three inflammatory disease groups and the status of the BMI (lower and higher) in relation to (Figure 2a) PCS12, (Figure 2b) MCS12 and (Figure 2c) metabolic age (mean ± SE). The groups studied were MI, LC and SLE. BMI, Body Mass Index; MCS12, Mental Component Summary; PCS12, Physical Component Summary.

### 3.5. Linear Multiple Regression of Inflammatory Variables and HRQoL

Of all the variables analyzed, the following lymphocytes, NLR, RDW, ESR, fibrinogen, PCS12 and MCS12 showed statistical associations under analytical regression runs (Table 3). The regression model was constructed using lymphocytes, NLR, RDW, ESR, fibrinogen, PCS12 and MCS12 as dependent variables and the age, sex, BMI, an interaction between disease and the BMI at different levels (lower and higher) as independent variables. An increase of one unit in BMI was associated with an average increase of 0.02 units in lymphocytes, while the interaction between SLE and a high BMI showed a decrease in lymphocytes by 0.72 units. Likewise, both the NLR and RDW showed an increase on average of 1.92 and 2.33 units, respectively, when both SLE and a high BMI were present at the same time.

On the other hand, the positive coefficients of 5.16 and 30.32 for the female sex evidence that women tend to have higher values in the ESR and fibrinogen, respectively, when compared to male individuals. In addition, the increase of one unit in the BMI was associated with an average increase of 0.54 and 3.62 units for both the ESR and fibrinogen, respectively (Table 3). In individuals who had both SLE and a high BMI, the ESR increased by 9.38 units and fibrinogen was increased by 141.39 units. These findings indicated that in individuals who had both SLE and a high BMI, the ESR and level of fibrinogen tend to be increasingly higher compared to those who only have one of these conditions or neither. However, if an individual has LC, the fibrinogen level tends to decrease by 52.89 units. This outcome stands that, in such individuals, the fibrinogen level tends to be lower compared to those who do not have LC. The negative coefficient for SLE showed that the fibrinogen level decreased by 67.91 units, which indicates fibrinogen tends to be lower in individuals with SLE compared to those who are without SLE.

Likewise, the physical and mental components also presented variations in their regression coefficients (Table 3). The presence of SLE was associated with an average decrease of 16.293 units in the PCS12 score and 10.71 units for MCS12. Likewise, the presence of LC was associated with a decrease of 11.13 units in the MCS12 score. These findings indicated that people who have LC tend to have a worse perceived quality of life compared to people who without LC, while in individuals who have both SLE and high BMI, the perceived quality of life trends to be significantly better compared to those who only have one of these conditions or none, since their regression coefficient increased by more than 13 units.

## 4. Discussion

This research aimed to characterize differential inflammatory responses and diagnostic tools considering the relationship between BMI and clinical and metabolic parameters in three inflammatory diseases. Anthropometric, biochemical, and quality of life parameters were measured in patients with MI, LC and SLE, presenting a relationship in the following significant variables: waist, total fat mass, visceral fat, metabolic age, PCS12 and MCS12, which could be associated with cardiovascular risk [59], inflammation [60] and quality of life [61]. On the other hand, LC showed significance in insulin, glucose, AST/ALT ratio, SBP and DBP, while SLE showed remarkable SBP and DBP, which are known cardiovascular risk factors determining health outcomes.

In this context, metabolic syndrome represents a significant public health problem [62], especially in obese and overweight individuals, who tend to have a low-grade proinflammatory state [63] that affects adipose tissue function [64]. Our results indicated that patients with MI showed worse anthropometric and biochemical impairments compared with the other two groups as expected, suggesting an increased risk of developing diseases such as cardiovascular disease, atherosclerosis and diabetes mellitus [59]. These conditions were associated with both an increased waist circumference, characteristic of metabolic syndrome [65], and BMI, both of which are recognized as key predictors of metabolic disorders [60]. The increased insulin levels observed in these patients may be related to insulin resistance [59], which triggers cellular stress and tissue dysfunctions that activate inflammatory cascades [26]. The impact of an elevated BMI on metabolic age was explained by factors such as body weight, fat percentage, physical activity and energy metabolism [52]. In addition, significant differences were found in scores of PCS12 and MCS12 components of HRQoL, confirming the influence of BMI on both [66]. The existing literature argues that obesity negatively affects HRQoL and that a higher degree of obesity is associated with greater impairment [61,67]. However, patients with metabolic inflammation in this study reported feeling slightly better both physically and mentally compared to the other groups tested.

On the other hand, LC, characterized by a persistence of symptoms after SARS-CoV-2 infection, is associated with an exacerbated inflammatory state and immune dysfunctions [11,68], which appear to be linked to a post-viral immune response [11,68]. This process is mediated by dysfunctional adipocytes that release proinflammatory cytokines, contributing to organ dysfunction and increasing the risk of viral complications [64]. In patients with LC and a high BMI, a clinical profile with higher blood pressure, triglycerides, glucose and AST/ALT ratio, in addition to other significant metabolic alterations, was observed. These findings were consistent with previous studies that have described abnormal metabolic profiles in patients with LC, characterized by imbalances in glycemic, lipid and inflammatory markers [69], suggesting an increased risk of cardiovascular events and liver dysfunction, in line with published studies [70,71]. Furthermore, obesity comorbidities and the severity of acute infection aggravate LC, affecting patients’ health [72]. This feature underscores the need to address risk factors such as obesity due to its impact on immunocompetence and endocrine metabolism [28]. This group of patients also showed the lowest levels of quality of life and a higher degree of sadness, highlighting the need for long-term follow-up in terms of both physical and mental health. This outcome is not only crucial to assess the social and economic impact of LC, but also to avoid an overload on healthcare systems [73,74].

In this context, SLE is an autoimmune disease characterized by prolonged systemic inflammation and multiple clinical manifestations [33,75,76], whose pathophysiology involves an exaggerated activation of B and T cells, which intensifies the immune response [75]. Our results showed that patients with SLE and a higher BMI had differences in clinical variables like those observed in patients with LC, along with increased SBP and DBP. These findings were consistent with previous studies indicating that higher adiposity is associated with a higher prevalence of and worse prognosis in immune-mediated diseases [27,77]. Recent data also reveal a high prevalence of overweight and obesity in SLE patients [5,78], and prospective studies suggest that the immune process in these patients has a greater impact on metabolic variables [79]. In addition, the burden of cardiovascular disease is high in these patients, partly due to risk factors such as hypertension, which is more common in people with SLE than in those without SLE [80,81]. Interestingly, SLE patients in our study showed a higher MCS12 score than other groups, suggesting that an elevated BMI may have a potential protective effect, although this hypothesis requires further investigation. Regarding PCS12, our results were consistent with previous studies reporting that elevated BMI is associated with worse HRQoL, particularly in the physical component, in patients with SLE [82]. Noteworthy, in most studies, the variable MCS12 has not been evaluated, despite being relevant as the third most weighted variable [83].

When performing the 3 × 2 ANOVA interaction analyses (Disease × BMI), it was found that variables such as waist circumference, total fat mass, metabolic age, SBP and MCS12 were dependent on both disease type and BMI, showing effect modification suggesting the need for differentiated clinical and pharmacological interventions, as such interactions could be related to cardiovascular risk, unhealthy lifestyles and systemic inflammation [52].

Understanding inflammatory processes is essential to address the underlying mechanisms and management of diseases such as LC and SLE, as they impact prognosis, treatment and outcomes [84]. The routine measurement of inflammatory markers, such as ESR, NLR, lymphocytes and fibrinogen, can provide valuable clinical information [85]. Our results show that patients with LC and a higher BMI have elevated levels of CRP, leukocytes, platelets, and ESR. These findings are consistent with previous studies reporting persistent elevations in inflammatory and metabolic markers, such as ferritin, hemoglobin, albumin, CRP, ESR and LDH, indicating a continued deterioration in these markers [86]. Contrariwise, patients with SLE exhibited the worst values for inflammatory variables and reduced lymphocyte levels, which could be associated with autoimmunity or therapeutic management [87]. These findings are consistent with investigations identifying differences in LDH, lymphocytes and ESR in SLE patients, particularly in relation to the BMI [27,88,89]. These findings underscore the importance of a comprehensive approach in the assessment of inflammatory markers to optimize and perform the management of these diseases.

Furthermore, an increased BMI was associated with higher uric acid levels in all three inflammatory states assessed. This finding is in accordance with the literature, which indicates that uric acid, an oxidative metabolite, is elevated in various inflammatory states [88,90,91] and is often associated with a dysfunctional lipid profile [79]. Obesity is a complex disease linked to an increase in several inflammatory markers [35], LC is associated with chronic low-grade inflammation [41] and SLE is characterized by chronic and generalized inflammation [45], which increases the risk of developing early atherosclerosis and cardiovascular problems throughout the disease, as indicated by recent guidelines [92]. More research is required to understand the underlying inflammatory mechanisms and the impact of the BMI on the regulation of systemic inflammation, which would allow the development of new treatments and prevention strategies [35,93,94,95].

In our study, we used two main methodological approaches: PP and ITT analysis. PP includes only patients who strictly followed the protocol, whereas ITT preserves the sample size and minimizes bias. The results showed that patients with SLE and a higher BMI had elevated levels of fibrinogen, RDW, ESR and NLR, indicating a direct relationship between an elevated BMI and systemic inflammatory status [96]. Elevated fibrinogen levels have been observed in several inflammatory diseases, including multiple sclerosis, Alzheimer’s disease, SLE and cancer [97], with the measurement of this marker being essential to assess and predict inflammatory and autoimmune comorbidities. In addition, an increased RDW has been associated with inflammation in cardiovascular disease, rheumatoid arthritis and SLE [98]. Although ESR is a useful measure, its specificity is limited. In contrast, the NLR is considered a more accurate indicator of systemic inflammation [99,100,101,102], correlating more consistently with inflammatory and clinical activity in several diseases, including SLE [50]. In autoimmune diseases such as SLE, there is a bidirectional link between inflammation and immune dysregulation. Chronic inflammation is not only a persistent symptom of these conditions, but also inflammatory processes exacerbate the immune response, creating a cycle that perpetuates autoimmune activity and increases the risk of complications [44]. For this reason, diverse inflammatory markers play a pivotal role in the assessment of inflammatory activity and relapse monitoring in SLE, as they allow the accurate measurement of the degree of inflammation and guide informed therapeutic decisions [34]. In addition, they provide crucial information to improve diagnosis, facilitate personalized treatment and improve the understand of the impaired immune response, which is key to proper management and accurate prognosis in these complex patients. Indeed, the analysis of inflammatory markers in SLE patients is useful to understand mutual interactions with the immune system.

In the HRQoL study, instruments such as the SF-12 questionnaire [21,22], widely used in patients with SLE, obesity, MetS and COVID-19, according to previous research [54,55,57,58], were used. In our study, the use of the SF-12 scale revealed that an elevated BMI negatively impacted PCS12 in all three diseases studied [103,104]. In patients with SLE, approximately one-third are overweight, which is generally associated with worsening symptoms, reduced functional capacity, and decreased quality of life [61,67,105]. However, our findings indicated that SLE patients with a high BMI showed an increase in the MCS12 score, an indicator of mental well-being, suggesting an unexpected protective effect. This phenomenon is remarkable, as a high BMI is expected to negatively impact quality of life [67,106]. One possible explanation for this increase in MCS12, despite having a high BMI, is the significant influence of age in these patients. We observed that those with a high BMI also tend to be older compared to their low BMI peers. This suggests that maturity and life experience may contribute to greater mental well-being, even when other factors, such as being overweight, may be present [55]. The results of the NUTRiMDEA study support this idea, showing that in people over 40 years of age with metabolic diseases, MCS12 is also increased, especially in patients with diabetes and hypertension [66]. In addition, a study of SLE patients over 60 years of age found that, although their physical quality of life declined with age, their mental well-being remained high, regardless of accumulated damage or disease activity [107]. This observation shows that while a high BMI is often associated with poorer quality of life, factors such as age and emotional maturity may exert a more significant influence on the mental well-being of SLE patients [82,108]. It is therefore crucial to consider how the interaction between BMI and age may affect mental quality of life in this patient group, which could inform more effective management and treatment strategies. Furthermore, a directly proportional relationship between a high BMI and metabolic age was observed in all inflammatory diseases, further suggesting that body weight might influence the inflammatory response [109]. These findings highlight the importance of assessing HRQoL including BMI, in the regulation of inflammation, while also considering metabolic age as a relevant factor.

Adjusted multiple regression analysis performance revealed that the coexistence of SLE and a high BMI amplifies the inflammatory effect, with a significant increase in markers such as the NLR, RDW, ESR and fibrinogen. These results suggest that the combination of SLE and an increased BMI potentiates systemic inflammation, which could have a negative impact on cardiovascular health and metabolism, in line with previous studies [44,110]. A decrease in lymphocyte levels was also identified in SLE patients with a high BMI, suggesting an altered immune response or increased inflammatory activity [111]. This finding is related to type I interferon activity and an increase in low-density granulocytes (LDG), factors that contribute to impaired quality of life in these patients [112]. Overall, the combination of SLE and elevated BMI more markedly affects inflammatory markers compared to other groups, highlighting the importance of these associations for improving the clinical management of this population.

The present study has some strengths that should be mentioned. First, validated, widely used marker scores have been used, which may facilitate future comparisons. In addition, three different chronic inflammatory states were included in the analysis, allowing for a factorial design to perform robust analyses with the recruited sample size. Moreover, the collection of validated anthropometric, biochemical, and quality of life data can provide a broader and comparable picture of the population in the face of the implementation of precision nutrition and medicine. However, this research has several limitations that must be considered, as a sequential recruitment led to an unequal distribution by sex. Although sex was used as an adjustment variable in the analysis, this disequilibrium could affect the generalizability of the findings. Furthermore, it was decided to stratify by the median BMI to ensure that subjects in each category were comparable and to produce balanced groups, although methodological biases associated with “per protocol” and “intention to treat” approaches cannot be ruled out. Also, another limitation is the use of a standard test to measure CRP instead of the high-sensitivity test (hs-CRP), which is more necessary to assess and monitor low-grade inflammation, which might have affected the interpretation of the inflammatory assessments. It is important to note that the sample sizes for certain groups are small, which may jeopardize the reliability and generalizability of some comparisons. This limitation should be acknowledged and analyzed in relation to the implications when interpreting results, as small sample sizes may lead to type I and type II errors. However, despite these constraints, the results are plausible and do not contradict established scientific principles, suggesting a reasonable basis for future research in the area.

## 5. Conclusions

This research highlighted the key role of BMI in shaping clinical outcomes in patients with MI, LC and SLE. A higher BMI was linked to worse clinical and inflammatory profiles, particularly in SLE patients, where an elevated BMI was observed with increased inflammation markers. These findings further highlight the critical importance of considering BMI in the management of inflammatory diseases.

Adapting interventions based on BMI can optimize treatment effectiveness and contribute to better long-term outcomes in patients with chronic inflammatory conditions.

## Figures and Tables

**Table 1:** Comparison of anthropometric measures, body composition, biochemical data and HRQoL between the three types of inflammatory diseases (MI, LC and SLE) and BMI status in the METAINFLAMMATION cohort.

		Metabolic Inflammation (MI)			Long COVID (LC)			Systemic Lupus Erythematosus (SLE)					
	Overall	Lower BMI	Higher BMI	*p* Test	Lower BMI	Higher BMI	*p* Test	Lower BMI	Higher BMI	*p* Test	Group of Disease	BMI Levels	Interaction
													ANOVA 3 × 2
**Variables**	**272**	**12**	**37**		**95**	**81**		**25**	**18**				
**Age (years)**	53 (10)	63 (9)	58 (11)	0.16	50(9)	52 (8)	0.25	48 (14)	58 (12)	**0.02**	**<0.001**	0.11	**<0.01**
**Gender = Woman (%)**	212 (77.9)	6 (50.0)	17 (45.9)	NA	81 (85.3)	64 (79.0)	0.37	23 (92.0)	17 (94.4)	NA	**<0.001**	0.36	0.81
**Metabolic age (years)**	56 (16)	58 (8)	70 (12)	**0.001**	44 (12)	65 (8)	**<0.001**	45 (17)	71 (12)	**<0.001**	**<0.001**	**<0.001**	**0.02**
**BMI (kg/m^2^)**	29.1 (5.8)	27.4 (1.1)	32.9 (3.4)	**<0.001**	24.3 (2.8)	34.1 (4.6)	**<0.001**	24.4 (2.3)	33.4 (3.6)	**<0.001**	**<0.001**	**<0.001**	**<0.01**
**Waist (cm)**	100.3 (15.2)	102.9 (5.7)	112.1 (10.9)	**0.001**	88.5 (9.4)	111.1 (12.0)	**<0.001**	87.9 (7.9)	109.5 (9.3)	**<0.001**	**<0.001**	**<0.001**	**<0.01**
**Total muscle mass (kg)**	47.2 (9.0)	50.9 (7.7)	54.9 (11.3)	0.2	43.5 (6.9)	50.0 (8.3)	**<0.001**	41.9 (5.0)	44.7 (7.4)	0.17	**<0.001**	**<0.001**	0.34
**Total fat mass (%)**	35.8 (8.7)	32.3 (4.5)	37.1 (6.7)	**0.01**	29.9 (6.5)	42.5 (7.2)	**<0.001**	31.4 (5.5)	44.1 (5.9)	**<0.001**	**0.64**	**<0.001**	**0.007**
**Visceral fat**	10.1 (5.0)	10.8 (3.2)	15.4 (4.9)	**0.001**	6.5 (2.4)	12.8 (4.0)	**<0.001**	6.4 (3.8)	12.5 (3.0)	**<0.001**	**<0.001**	**<0.001**	0.42
**Glucose (mg/dL)**	96 (15.7)	108 (19.8)	105 (17.7)	0.76	91 (7.5)	99 (19.7)	**0.001**	89 (7.6)	93 (17.3)	0.45	**<0.001**	**<0.01**	0.18
**Total cholesterol (mg/dL)**	190.7 (35.0)	183.2(26.7)	180.7 (35.0)	0.82	201.7 (35.8)	191.9 (29.9)	0.06	175.6 (34.4)	156.7 (33.6)	0.13	**<0.001**	**<0.05**	0.62
**Triglycerides (mg/dL)**	106.7 (56.2)	118.5 (49.5)	140.8 (46.9)	0.23	88.2 (38.8)	117.7 (59.0)	**<0.001**	109.2 (102.0)	109.7 (44.7)	0.99	**<0.05**	**<0.01**	0.38
**Insulin (μUI/mL)**	11.3 (11.3)	7.0 (2.5)	15.9 (11.2)	**0.004**	7.2 (4.7)	15.2 (15.5)	**<0.001**	8.6 (6.9)	15.1 (11.7)	0.11	0.63	**<0.001**	0.92
**AST/ALT ratio**	1.0 (0.3)	0.9 (0.3)	0.8 (0.1)	0.18	1.1 (0.3)	0.9 (0.2)	**<0.001**	1.2 (0.3)	1.1 (0.5)	0.75	**<0.01**	**<0.001**	0.45
**Transferrin (mg/dL)**	249.8 (38.5)	233.2 (77.8)	257.3 (35.9)	0.35	248.3 (32.2)	257.8 (39.2)	0.11	233.4 (36.2)	244.4 (29.9)	0.36	0.21	**<0.05**	0.63
**Prothrombin activity (%)**	103.1 (21.1)	105.6 (20.2)	108.4 (19.1)	0.7	107.3 (14.6)	101.5 (21.6)	0.06	96.7 (20.8)	85.5 (40.3)	0.36	**<0.01**	0.06	0.4
**SBP (mmHg)**	127.4 (19.2)	142.9 (9.0)	139.0 (21.8)	0.38	119.0 (17.4)	130.4 (16.0)	**<0.001**	120.5 (13.3)	138.8 (20.4)	**0.003**	**<0.001**	**<0.001**	**0.01**
**DBP (mmHg)**	77.0 (11.9)	80.8 (14.2)	81.3 (12.1)	0.9	73.8 (11.8)	80.0 (10.1)	**<0.001**	71.6 (11.9)	79.2 (11.6)	**0.04**	**<0.01**	**<0.001**	0.33
**Sadness = Yes (%)**	164 (60.1)	4 (33.3)	12 (32.4)	NA	65 (68.4)	63 (77.8)	0.22	11 (44.0)	7 (38.9)	0.98	**<0.001**	0.34	0.6
**PCS12**	36.0 (11.8)	49.9 (8.0)	45.4 (9.4)	**<0.001**	33.5 (9.3)	29.4 (8.8)	**<0.001**	44.0 (14.2)	36.5 (12.2)	**<0.001**	**<0.001**	**<0.001**	0.59
**MCS12**	40.2 (12.9)	52.1 (8.2)	46.5 (10.3)	**<0.001**	38.6 (12.6)	36.6 (13.2)	**<0.001**	38.6 (12.3)	47.3 (11.6)	**<0.001**	**<0.001**	0.62	**<0.05**

Data presented as mean ($\bar{x}$), standard deviation (SD), and *p* values. The significance threshold was set at *p* < 0.05, *t*-test was used to compare the mean of continuous variables and Chi-square (χ^2^) to compare categorical variables. The groups studied were MI, LC and SLE. Lower BMI refers to BMI < 28.7 kg/m^2^ and higher BMI refers to BMI > 28.7 kg/m^2^. Group column is the comparison of variables’ mean between the three inflammatory diseases (without considering BMI status). BMI levels are the comparison of variables’ mean between the lower and higher BMI. Interaction column means the *p* value of comparison between the three disease groups and the BMI levels (ANOVA 3 × 2). AST/ALT ratio, aspartate aminotransferase/alanine transaminase ratio; BMI, Body Mass Index; DBP, diastolic blood pressure; LC, long COVID; MCS12, Mental Component Summary; MI, metabolic inflammation; NA, not assigned; PCS12, Physical Component Summary; SBP, systolic blood pressure; SLE, systemic lupus erythematosus.

**Table 2:** Inflammatory status characteristics of METAINFLAMMATION study participants stratified by lower and higher BMI between the three types of inflammatory diseases (MI, LC and SLE).

		Metabolic Inflammation (MI)			Long COVID (LC)			Systemic Lupus Erythematosus (SLE)					
	Overall	Lower BMI	Higher BMI	*p* Test	Lower BMI	Higher BMI	*p* Test	Lower BMI	Higher BMI	*p* Test	Group of Disease	BMI Levels	Interaction
													ANOVA 3 × 2
**Variables**	**272**	**12**	**37**		**95**	**81**		**25**	**18**				
**Uric Acid (mg/dL)**	5.0 (1.3)	4.6 (1.0)	6.6 (1.3)	**<0.001**	4.6 (1.1)	5.4 (1.2)	**<0.001**	4.3 (1.1)	5.2 (1.3)	**0.04**	**<0.001**	**<0.001**	**<0.05**
**LDH (U/L)**	175.8 (33.4)	172.8 (28.8)	189.4 (42.8)	0.2	167.1 (28.2)	180.3 (34.4)	**0.01**	166.7 (31.1)	198.0 (30.8)	**0.008**	0.21	**<0.001**	0.35
**NT proBNP (pg/mL)**	60.9 (52.0)	83.0 (83.6)	43.5 (31.1)	0.16	66.4 (63.0)	50.8 (33.7)	0.05	59.9 (38.1)	74.0 (39.7)	0.36	0.23	**0.02**	0.15
**C-reactive protein (mg/L)**	3.6 (6.1)	3.2 (3.4)	3.7 (3.9)	0.67	2.0 (3.0)	3.8 (4.1)	**0.003**	5.5 (14.2)	10.3 (10.6)	0.28	**<0.001**	**<0.01**	0.3
**IL-6 (pg/mL)**	3.4 (2.4)	3.8 (2.4)	3.1 (1.3)	0.37	3.3 (3.1)	3.2 (1.5)	0.72	3.8 (2.8)	3.8 (1.6)	0.94	0.76	0.56	0.8
**Ferritin (ng/mL)**	107.3 (97.1)	168.5 (121.3)	157.0 (132.0)	0.81	96.0 (82.7)	105.3 (100.5)	0.53	89.9 (89.8)	97.4 (57.9)	0.78	**<0.01**	0.63	0.86
**Leukocytes (10 × 10^3^/microL)**	6.2 (1.8)	5.6 (1.3)	6.7 (1.8)	0.06	5.8 (1.7)	6.5 (1.6)	**0.02**	6.2 (2.2)	6.3 (3.3)	0.95	0.81	**<0.05**	0.56
**Lymphocytes (10 × 10^3^/microL)**	1.9 (0.6)	1.7 (0.2)	1.9 (0.7)	0.26	1.9 (0.6)	2.0 (0.5)	0.06	1.9 (0.7)	1.4 (0.6)	**0.04**	**<0.05**	0.371	**<0.05**
**NLR**	2.1 (1.8)	2.0 (0.6)	2.3 (0.7)	0.13	1.9 (0.7)	2.0 (0.9)	0.38	2.1 (1.3)	4.4 (6.1)	0.17	**<0.01**	**<0.05**	**<0.01**
**Platelets (10 × 10^3^/microL)**	256.1 (65.0)	229.6 (45.5)	246.0 (56.5)	0.38	252.8 (61.5)	275.5 (62.0)	**0.02**	247.2 (80.5)	234.2 (89.4)	0.68	0.09	0.06	0.37
**RDW (%)**	13.7 (2.3)	13.9 (0.8)	13.7 (0.5)	0.49	13.3 (0.6)	13.7 (3.0)	0.24	13.7 (0.8)	16.1 (5.4)	0.12	**<0.05**	**<0.05**	0.05
**ESR (mm)**	11.4 (8.3)	8.7 (7.9)	9.8 (8.6)	0.74	9.8 (6.5)	12.2 (7.2)	**0.03**	9.8 (8.4)	22.3 (12.9)	**0.004**	**<0.01**	**<0.001**	**<0.01**
**Fibrinogen (mg/dL)**	358.2 (85.2)	368.6(84.5)	383.1 (94.5)	0.99	334.0 (70.0)	364.5 (62.8)	**0.005**	321.2 (95.8)	457.4 (128.1)	**0.001**	**<0.01**	**<0.001**	**<0.001**
**D-dimer (ng/mL)**	353.3 (311.9)	315.5 (100.5)	342.1 (239.0)	0.68	352.9 (381.5)	371.4 (297.7)	0.74	331.5 (231.3)	367.5 (227.5)	0.68	0.85	0.62	0.99

Data presented as mean ($\bar{x}$), standard deviation (SD), and *p* values. The significance threshold was set at *p* < 0.05, *t*-test was used to compare the mean of continuous variables and Chi-square (χ^2^) to compare categorical variables. The groups studied were MI, LC and SLE, Lower BMI refers to BMI < 28.7 kg/m^2^ and higher BMI refers to BMI > 28.7 kg/m^2^. Group column is the comparison of variables’ mean between the three inflammatory diseases (without considering BMI status). BMI levels are the comparison of variables’ mean between the lower and higher BMI. Interaction column means the *p* value of comparison between the three disease groups and the BMI levels (ANOVA 3 × 2). ESR, erythrocyte sedimentation rate; IL-6, interleukin-6; LC, long COVID; LDH, lactate dehydrogenase; MI, metabolic inflammation; NLR, neutrophil/lymphocyte ratio; NT proBNP, N-terminal pro-B-type natriuretic peptide; RDW, red cell blood distribution Width; SLE, systemic lupus erythematosus.

**Table 3:** Linear multiple regression model for the variables: lymphocytes, NLR, ESR, fibrinogen, PCS12 and MCS12 adjusted for age, sex, BMI and the interaction between disease and BMI at different levels (lower and higher) on the METAINFLAMMATION cohort.

Dependent Variables	Independent Variables	Regression Coefficient (β)	CI (95%)	*p* Value
Lymphocytes (10 × 10^3^/microL)	BMI	0.02	0.0001 to 0.04	0.05
	SLE * Higher BMI	−0.72	−1.31 to 3.74	0.01
NLR	SLE * Higher BMI	1.92	0.11 to 3.74	0.04
RDW (%)	SLE * Higher BMI	2.33	0.03 to 4.63	0.05
ESR (mm)	Gender (woman)	5.16	2.66 to 7.66	<0.001
	BMI	0.54	0.25 to 0.83	<0.001
	SLE * Higher BMI	9.38	1.86 to 16.9	0.01
Fibrinogen (mg/dL)	Gender (woman)	30.32	4.21 to 56.42	0.02
	BMI	3.62	0.59 to 6.64	0.02
	Long COVID	−52.89	−104.22 to −1.56	0.04
	SLE	−67.91	−129.51 to −6.30	0.03
	SLE * Higher BMI	141.39	61.69 to 221.08	<0.001
PCS12	SLE	−16.29	−22.57 to −10.02	<0.001
MCS12	Long COVID	−11.13	−18.90 to −3.36	0.01
	SLE	−10.71	−19.60 to −1.80	0.02
	SLE * Higher BMI	13	1.79 to 24.21	0.02

Data were presented as regression coefficients (β), confidence intervals (95%) and *p* values. Adjusted for age, sex, BMI and for the interaction between disease and dichotomized BMI. BMI, Body Mass Index; CI, confidence interval; ESR, erythrocyte sedimentation rate; MCS12, Mental Component Summary; NLR, neutrophil/lymphocyte ratio; PCS12, Physical Component Summary; RDW, red cell blood distribution width.

## Data Availability

The data sets used and/or analyzed during the current study are available from the corresponding author on reasonable.
